# Molecular analysis of capsid protein of Homalodisca coagulata Virus-1, a new leafhopper-infecting virus from the glassy-winged sharpshooter, Homalodisca coagulata

**DOI:** 10.1673/2006_06_28.1

**Published:** 2006-10-18

**Authors:** W. B. Hunter, C. S. Katsar, J. X. Chaparro

**Affiliations:** United States Department of Agriculture, Agricultural Research Service, U.S. Horticultural Research Laboratory, Fort Pierce, FL 34945

**Keywords:** Cicadellidae, Dicistroviridae, Hemiptera, HoCV-1, Insect, Picorna, ssRNA

## Abstract

A new virus that infects and causes increased mortality in leafhoppers was isolated from the glassy-winged sharpshooter, Homalodisca coagulata (Say) (Hemiptera: Cicadellidae). The virus, named Homalodisca coagulata virus -1, HoCV-1, was associated with increased mortality of cultured 5^th^ instar H. coagulata. To identify the presence of H. coagulata viral pathogens, cDNA expression libraries were made from adult and nymphs. Analysis using reverse transcriptase PCR demonstrated that the virus was present in midgut tissues. As the viral capsid proteins are commonly used in classification of newly discovered viruses, the capsid proteins (CP) of the virus discovered in H. coagulata was examined. The order of the polyprotein subunits of HoCV-1 capsid proteins was determined to be CP2, CP4, CP3, and CP1. The CP4/CP3 (AFGL/GKPK) cleavage boundary site was clearly identified when the sequences were aligned. The putative CP3/CP1 (ADVQ/SAFA) cleavage site and the putative CP2/CP4 (VTMQ/EQSA) cleavage site of HoCV-1, respectively, were located in the same region as that of the other viruses. After alignment, the CP3/CP1 cleavage sites and CP2/CP4 cleavage sites of the viruses analyzed fell within 50 amino acids of one another. As with the cricket paralysis virus, HoCV-1 was found to be mainly comprised of β-sandwiches in CP1-3 with a jelly roll topological motif. CP4 of HoCV-1 appeared to be mainly α-helical in structure. CP1-4 domains are most homologous to insect picorna-like virus coat proteins as was demonstrated by the results of the BLASTP and PSI-BLAST tests, and is strongly supported by the structural modeling. While sequence homology between the cricket paralysis virus and HoCV-1 was low, the global structure of the proteins was conserved. Sequence identities were analyzed by in silico comparison to known genes in the public database, NCBI. Phylogenetic analysis performed using the optimized protein alignment generated a phylogram containing 5 clades. Clade 1 consisted of Drosophila C virus, Clade 2 consisted of cricket paralysis virus, Clade 3 of Triatoma virus, Plautia stali intestine virus, Himetobi P virus, black queen cell virus, and HoCV-1. Clade 4 encompassed acute bee paralysis virus and Kashmir bee virus, and Clade 5 consisted of Rhopalosiphum padi virus. Analysis of the capsid protein of this new leafhopper virus provided significant evidence that it is related to other ssRNA insect viruses within the Family, Dicistroviridae. The HoCV-1, capsid protein sequence has been deposited in GenBank, Accession number: DQ308403.

## Introduction

Few viral pathogens are known from leafhoppers. The glassy-winged sharpshooter, Homalodisca coagulata (Say) (Hemiptera: Cicadellidae) is the primary vector of Pierce’s disease of grapes, which is caused by the bacterial plant pathogen, Xylella fastidiosa. There are numerous plant diseases caused by the Xylella bacteria which are known as ‘Scorch’ diseases due to the dry leaf appearance in susceptible plants. In grapes, susceptible plants have reduced yields and may die after infection thus causing severe economic losses. Research to examine the interactions between H. coagulata and Xylella requires mass rearing of large numbers of H. coagulata for use in transmission experiments. Attempts to mass rear H. coagulata, resulted in an observable increase in mortality during the 5^th^ instar stage (personal observations) making it difficult to produce adult H. coagulata for research needs. Mortality in cultured insects is often due to the presence of undetected insect pathogens. Insect cell cultures have been used to detect insect pathogens (Hunter et al. 2001, Funk et al. 2001). Our approach was to identify the presence of H. coagulata viral pathogens, using cDNA expression libraries, which provide a overview of the transcripts in adults and nymphs. As viral capsid protein (CP) sequences, are commonly used in classification of newly discovered viruses we chose to elucidate the CP of the virus discovered in H. coagulata. Sequence identities were analyzed by comparison to known genes in the public database, NCBI. Viral sequences which were identified were then validated by amplification and bidirectional sequencing. A new viral pathogen of the H. coagulata was identified and shown to be significantly related to insect viruses within the Family, Dicistroviridae. The virus herein was named: Homalodisca coagulata virus–1, HoCV-1.

## Materials and Methods

### cDNA library construction

A whole body, adult cDNA library was made from 160 adult H. coagulata, and a 5^th^ instar cDNA library was made from 140 nymphs collected from citrus groves near Riverside, California. Insects were collected into liquid nitrogen and total RNA subsequently extracted using the guanidinium salt-phenol-chloroform procedure as described by Strommer (1993). Contaminating DNA was removed using RQ1 RNase-free DNase (Promega, www.promega.com) and poly(A)+ RNA was purified using a MicroPoly(A)Pure™ kit (Ambion, Inc., www.ambion.com) following the manufacturer’s instructions. A directional cDNA library was constructed in Lambda Uni-ZAP^®^ XR Vector using Stratagene’s ZAP-cDNA Synthesis kit (Stratagene, www.stratagene.com). The resulting DNA was packaged into lambda particles using Gigapack^®^ III Gold Packaging Extract (Stratagene). Mass excision of the amplified library was carried out using Ex-Assist^®^ helper phage (Stratagene). An aliquot of the excised, amplified library was used for infecting XL1-Blue MRF’ cells and subsequently plated on LB agar containing 100 μg/ml ampicillin. Bacterial clones containing excised pBluescript SK(+) phagemids were recovered by random colony selection. pBluescript SK(+) phagemids were grown overnight at 37°C and 240 rpm in 96-deep well culture plates containing 1.7 ml of LB broth, supplemented with 100 μg/ml ampicillin. Archived stocks were prepared from the cell cultures using 75 μl of a LB-amp, glycerol mixture and 75 μl of cells. These archived stocks are held at the U.S. Horticultural Research Laboratory where they are kept in an ultra low temperature freezer (−80° C). Plasmid DNA was extracted using the Qiagen 9600 liquid handling robot and the QIAprep 96 Turbo miniprep kit according to the recommended protocol (QIAGEN, www.qiagen.com). Bidirectional sequencing of HoCV-1 clones were completed using T3 and T7 primers, sequencing reactions were performed using the ABI PRISM^®^ BigDye^TM^ Primer Cycle Sequencing Kit (Applied Biosystems, www.appliedbiosystems.com) along with a universal T3 primer. Sequencing reaction products were precipitated with 70% isopropanol, resuspended in 15 μl of sterile water and loaded onto an ABI 3730xl DNA Analyzer (Applied Biosystems). Two other H. coagulata cDNA libraries (salivary gland and midgut) were also examined for virus sequences.

### Sample preparation and rt-PCR analysis

Midgut and salivary gland tissues from approximately 40 H. coagulata adults, collected near Riverside, California, were tested for presence of HoCV-1, using reverse transcriptase PCR (rtPCR), with the amplicons sequenced and compared using Blast analyses, NCBI database to identify homologous viral sequences. Upon arrival at the laboratory, H. coagulata individuals were removed from RNA *late*r^®^ and washed in 1X phosphate buffered saline (PBS), pH 7.0 at 4°C. Tissues from whole insects were dissected in cold 1X PBS, pH 7.0. Samples were ground directly in Buffer RLT (Qiagen) using sterile plastic pestles. RNA extractions were performed using the RNeasy^®^ Mini Kit (Qiagen) following the manufacturer’s instructions. Total RNA was eluted in RNase-free water and quantified by UV spectrophotometry. An equal concentration (500 ng) of total RNA was retrotranscribed for each sample reaction by first combining RNA, 1 μl dNTPs (10mM), and 1 μl oligo(dT)_17_ primer (2.0 μg/μl) for a reaction volume of 13 μl. The mixture was incubated at 65°C for 5 min after which, 4 μl 5x buffer, 2 μl dithiothreitol (DTT), 1 μl RNasin^®^ ribonuclease inhibitor (40 U/μl) (Promega), and 1 μl SuperScript^TM^ III (200 U/μl) (Invitrogen) were added. The reaction mixture was then incubated at 55°C for 1 hr and subsequently terminated at 65°C for 10 min. Primers were designed using Primer3 (Rozen and Skaletsky 2000) GenBank^®^ accession number DQ308403 (Homalodisca coagulata virus-1 capsid protein). Amplification was performed using 22.5 μl Platinum^®^ PCR Supermix (Invitrogen), 1 μl of the forward (5′-TCC GAG TTC TCA GCC AAA CT-3′) and reverse (5′-CGG CAT ATC GAA ATG AGG TT-3′) primers combined (10 μM) each, and 1.5 μl cDNA. The reaction mixture was subjected to an initial denaturation at 95°C for 2 min followed by 35 cycles of 95°C for 30 sec, 60°C for 30 sec, and 72°C for 1 min, and concluded with a final DNA extension at 72°C for 5 min. Validation of five amplicons by sequencing were completed, after which samples were considered positive when a visible amplicon (443 nucleotides) was present after separation on a 1 % agarose (TAE) gel stained with ethidium-bromide, EtBr (0.5 μg/ml).

### Computer analysis of HoCV-1 nucleic acid and deduced protein sequences

Base confidence scores were designated using TraceTuner^®^ (Paracel, www.paracel.com). Low-quality bases (confidence score <20) were trimmed from both ends of sequences. All quality trimming, vector trimming and sequence fragment alignments were executed using Sequencher^®^ software (Gene Codes Corp., www.genecodes.com). Amplicons were sequenced and compared by Blast analyses for adults and midgut and salivary gland tissues that tested positive for HoCV-1 by rtPCR to identify homologous viral sequences.

The potential status of the of the HoCV-1 capsid protein was determined based on Blast homology searches using the National Center for Biotechnology Information Blast server (http://www.ncbi.nlm.nih.gov) with the sequence comparisons made to protein databases (BLASTX, TBLASTX, BLASTP). Contig assembly parameters were set using a minimum overlap of 50 bases and 90% identity match. Multiple alignments were performed with CLUSTAL X, version 1.83 (Thompson et al. 1997) using the following sequences (with their respective GenBank^®^ accession numbers): ABPV (NC_002548), BQCV (NC_003784), CrPV (NC_003924), DCV (NC_001834), HiPV (NC_003782), KBV (NC_004807), PSIV (NC_003779), RhPV (NC_001874), TrV (NC_003783). Protein molecular weights were approximated using suite 2 of the Stothard method for sequence manipulation (Stothard 2000). Phylogenetic trees were constructed using the capsid protein, CP, sequences via the neighbor-joining method using PAUP* version 4.0 (Swofford 2003). For each tree, confidence levels were estimated using the bootstrap resampling procedure (2000 replications).

### Theoretical modeling of the HoCV-1 capsid proteins

The theoretical structures of HoCV-1, CP1-4 were determined using CrPV, protein database, PDB, entries 1b35a, 1b35b, 1b35c and 1b35d respectively. The theoretical structures of HoCV-1 CP 1–4 were numbered according to the standard convention, with amino acids numbered starting from 001 in each protein.

### Computer analysis of EST sequences

Vector and quality trimming, base quality scores generated with TraceTuner^®^ (Paracel, www.paracel.com) and sequence fragment alignments were executed using Sequencher^®^ software (Gene Codes, www.genecodes.com). Sequence corresponding to vector contaminants was removed from the dataset. To estimate the number of genes represented in the library and the redundancy of specific genes, ESTs were assembled into contigs using Sequencher^®^. Contig assembly parameters that were set using a minimum overlap of 50 bases and 95% identity match.

Standard DNA and protein sequence analyses performed used BLASTn and BLASTp, respectively (Altschul et al. 1990 1997). The National Center for Biotechnology Information (NCBI) biosynthesis databases were searched via the Entrez Search and Retrieval system (http://www.ncbi.nlm.nih.gov/gquery/gquery.fcgi). The theoretical molecular weight and pI value of the predicted protein was calculated by Compute PI/MW on the ExPASy server (http://au.expasy.org). Compute pI/MW calculates the molecular weight of an input sequence by adding the average isotopic mass of each amino acid plus one water molecule.

Structural modeling was performed using the Robetta automated server (http://robetta.bakerlab.org/). Robetta is a full-chain protein structure prediction server and permits both initial and comparative models of protein domains. In addition, Robetta performs domain parsing and 3-D modeling included fragment library generation. The programs utilize the Ginzu domain parsing and fold detection method and the Rosetta fragment insertion method (Bonneau et al. 2002, Chivian et al. 2003, Kim et al. 2004, Simons et al. 1997). Protein chains were scanned to identify homologs using PDB-BLAST, FFAS03, 3D-Jury, and the Pfam-A protein family database. PSI-BLAST multiple sequence alignment then assigns regions of increased likelihood of including an adjoining domain using sequence clusters (Bowers et al. 2000; Rohl and Baker 2002). Graphic rendering of the predicted 3-D structures were done using the PDB file created by Robetta and Protein Explorer (http://www.molvis.sdsc.edu/protexpl/frntdoor.htm).

### Phylogenetic analyses

Multiple sequence alignments of predicted HoCV-1 capsid protein amino acid sequences (1–4) were performed using CLUSTAL X version 1.81 algorithm (Thompson et al. 1997). Guide trees were generated using neighbor-joining and Bayesian methods. Neighbor-joining analyses were performed using PAUP* version 4.0β10 (Swofford 2003). Neighbor joining estimates of the desaturase phylogenies were obtained by estimating distance parameters in PAUP* (Swofford 2003). Neighbor-joining bootstrap analyses of 2000 replicates were performed on each data set using a heuristic search to identify the most optimal tree. Analyses were unrooted.

## RESULTS

**Figure 1:**
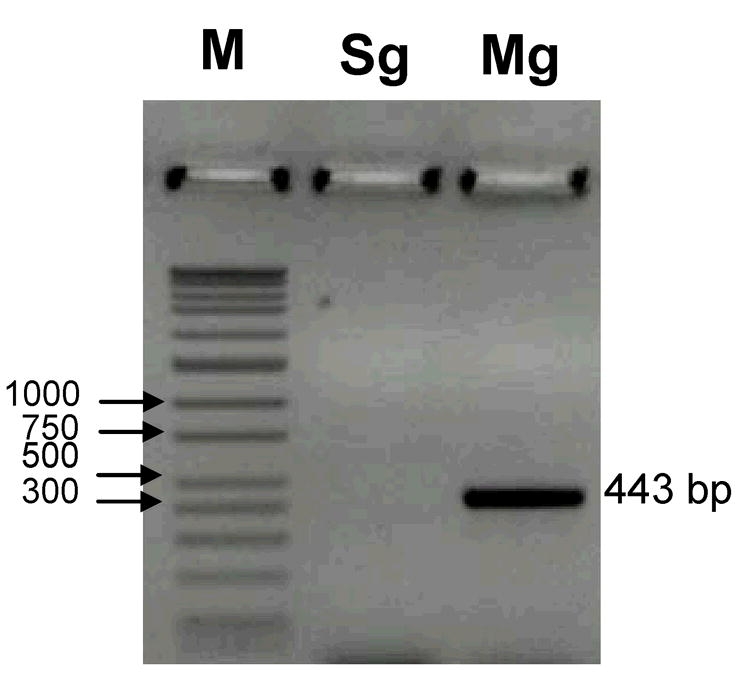
Gel of detected virus from salivary gland (Sg) and midgut (Mg) tissues of Homalodisca coagulata adults tested for presence of HoCV-1, using rtPCR. Both types of tissues from individual insects were dissected and analyzed in a pairwise fashion. Only midgut tissues were shown to test positively for virus presence. M = ladder wide-range DNA marker (16 fragments 50-10,000 bp), Sg = salivary glands, Mg = midgut tissue. Amplified fragment ~ 443 bp, was validated by sequencing of the amplified product and Blast analysis.

A total of 8,600 random EST sequences were generated from H. coagulata. Viral sequences were initially discovered through analysis of the cDNA library generated from whole bodies of adults. Approximately 5.8% (500) of the clones were homologous to viral sequences as indicated by BLASTX results. Analysis using rtPCR showed that the virus was in the midgut tissues, but not in salivary glands (Fig. 1). Blast analysis of the viral capsid protein indicated that the amino acid sequence had homology to the capsid proteins of insect picorna-like viruses, within the Family Dicistroviridae. The capsid protein sequence of HoCV-1 was submitted into GenBank (accession number: DQ308403), and was used to perform TBLASTX search of the NCBI database. Sequences with significant amino acid identity were downloaded for comparison to the HoCV-1 sequence. Comparisons were made to capsid protein sequences of: cricket paralysis virus (CrPV) [NC_003924], Drosophila C virus (DCV) [NC_001834], Triatoma virus (TrV) [NC_003783], Himetobi P virus (HiPV) [NC_003782], Plautia stali intestine virus (PSIV) [NC_003779], black queen cell virus (NC_003784), acute bee paralysis virus (ABPV) (NC_002548), Kashmir bee virus (KBV) [NC_004807], and Rhopalosiphum padi virus (RhPV) [NC_001874] (Table 1).

**Table 1:**
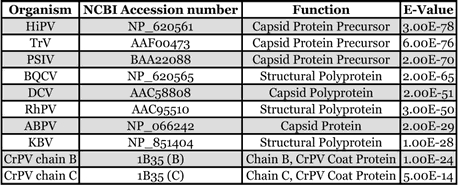


**Figure 2:**
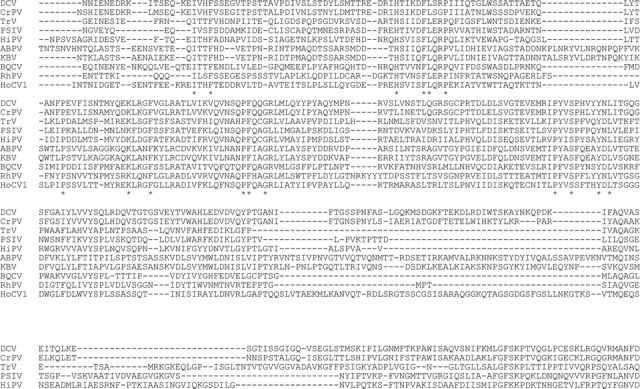
Multiple alignment of predicted amino acid sequence of putative capsid proteins of Homalodisca coagulata virus-1 (HoCV-1) with the homologous proteins of Drosophila C virus (DCV) (Johnson and Christian 1998), cricket paralysis virus (CrPV) (Koonin and Gorbalenya 1992), Triatoma virus (TrV) (Czibener et al. 2000), Plautia stali virus (PSIV) (Sasaki et al. 1998), Himetobi P virus (HiPV) (Nakashima et al. 1999), acute bee paralysis virus (ABPV) (Govan et al. 2000), Kashmir bee virus (KBV)( De Miranda et al. 2004), black queen cell virus (BQCV) (Leat et al. 2000), Rhopalosiphum padi virus (RhPV) (Moon et al. 1998),. Predicted cleavage sites are highlighted in yellow, areas of identity in grey shading (*) denotes conserved amino acids.

The HoCV-1 capsid protein had a predicted molecular weight of 97.94 kD and a pI of 5.69 (http://us.expasy.org/cgibin/pi_tool). The comparison of the predicted amino acid sequence of putative HoCV-1 capsid protein identified several clusters of conserved amino acids (Fig. 2) separated by large regions of low, or no homology, or gaps. Amino acid identity between CP1-4 of CrPV and HoCV-1 ranged from less than 20% for CP1 and CP4, to 35% for CP2 (Table 2).

**Table 2:**
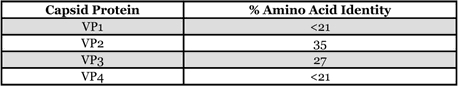


**Figure 3:**
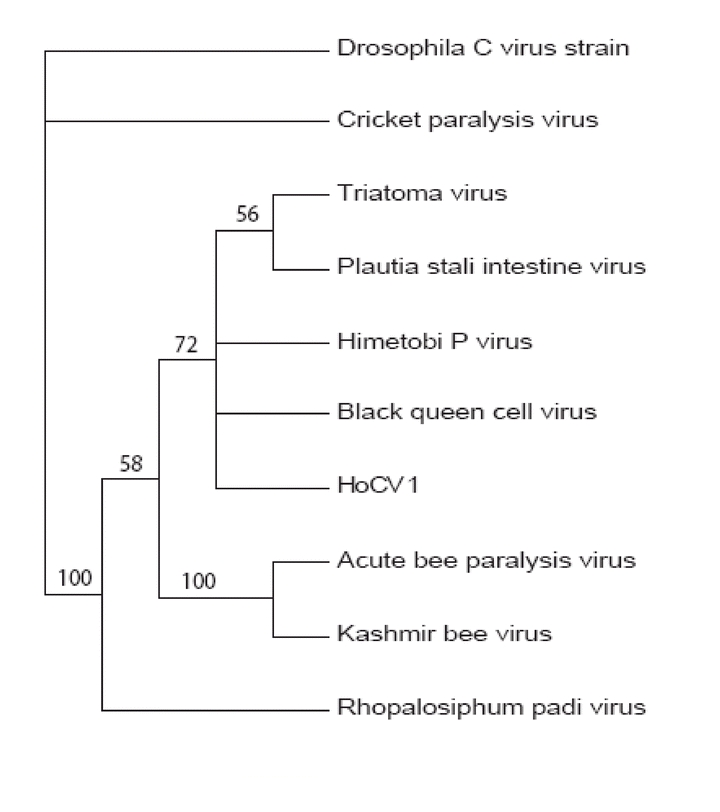
Neighbor-joining analyses of Homalodisca coagulata virus-1, HoCV-1 capsid protein amino acid sequence with selected Cripavirus members: Drosophila C virus (DCV) (Johnson and Christian 1998), cricket paralysis virus (CrPV) (Koonin and Gorbalenya 1992), Triatoma virus (TrV) (Czibener et al. 2000), Plautia stali virus (PSIV) (Sasaki et al. 1998), Himetobi P virus (HiPV) (Nakashima et al. 1999), black queen cell virus (BQCV) (Leat et al. 2000), acute bee paralysis virus (ABPV) (Govan et al. 2000), Kashmir bee virus (KBV)( De Miranda et al. 2004), Rhopalosiphum padi virus (RhPV) (Moon et al. 1998). Phylogenetic trees were constructed via the neighbor-joining method using PAUP* version 4.0 (Swofford 2003). For each tree, confidence levels were estimated using the bootstrap resampling procedure (2000 replications).

The phylogenetic analysis performed using the optimized protein alignment generated a phylogram containing 5 clades (Fig. 3). Clade 1 consisted of Drosophila C virus, Clade 2 consisted of c ricket paralysis virus, Clade 3 of Triatoma virus, Plautia stali intestine virus, Himetobi P virus, black queen cell virus, and HoCV-1. Clade 4 encompassed Acute bee paralysis virus and Kashmir bee virus, and Clade 5 consisted of Rhopalosiphum padi virus. When the four capsid polyprotein units were analyzed individually HoCV-1 always fell within the same clade (Fig. 3) as Triatoma virus, Plautia stali intestine virus, Himetobi P virus, and black queen cell virus.

**Figure 4:**
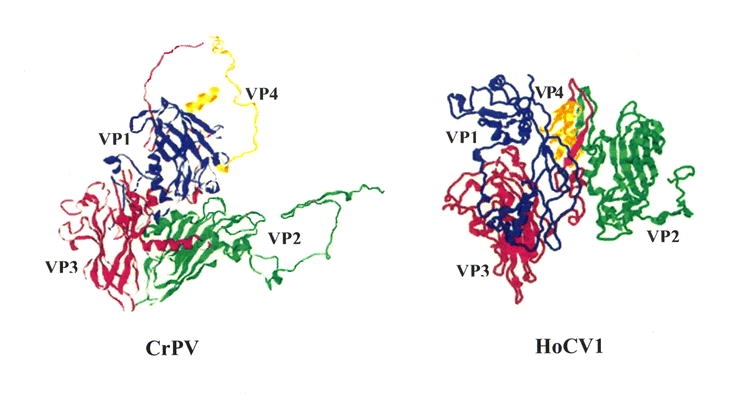
Capsid protein structural prediction and model comparison of Homalodisca coagulata virus-1, HoCV-1 to the Cripavirus type member, cricket paralysis virus, CrPV, Family Dicistroviridae.

The amino acid sequence alignment was scanned for putative cleavage sites of the capsid polyprotein (Table 3). Viruses in this group typically have three cleavage sites (van Munster et al. 2002). The order of the polyprotein subunits is CP2, CP4, CP3, CP1. The CP4/CP3 (AFGL/GKPK) cleavage boundary site was clearly aligned with all the aligned viruses. The putative CP3/CP1 (ADVQ/SAFA) site and the putative CP2/CP4 (VTMQ/EQSA) cleavage site of HoCV-1, respectively, were located in the same region as that of the other viruses. After alignment, the CP3/CP1 cleavage sites and CP2/CP4 cleavage sites of all viruses analyzed fell within 50 amino acids of one another (Fig. 2). Putative cleavage sites remain to be validated by n-terminus sequencing. As with CrPV, HoCV-1 was found to be mainly comprised of β-sandwiches in structure in CP1-3 with a jelly roll topological motif (http://pdbbeta.rcsb.org/pdb/explore.do?structureId=1b35) (Bonneau et al. 2002). There were few secondary structures found in CP4 of both CrPV and HoCV-1. CP4 of HoCV-1 appeared to be mainly α-helical in structure. CP1-4 domains are most homologous to insect picorna-like virus capsid proteins as demonstrated by the BLASTP and PSI-BLAST results, as well as by the structural modeling. While sequence homology between CrPV and HoCV-1 was low (Table 2, Fig. 2) the global structure of the proteins was conserved (Fig. 4).

**Table 3:**
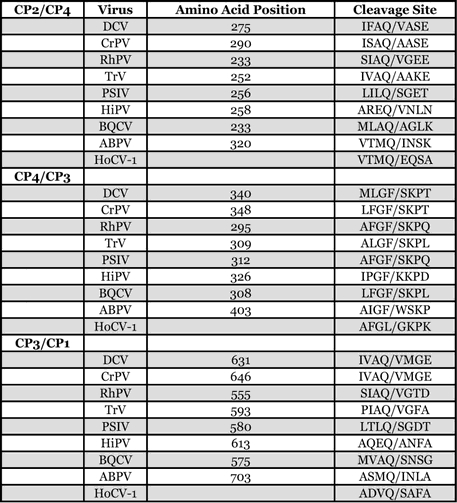


## DISCUSSION

Analysis of the HoCV-1 capsid protein demonstrated that the virus is taxonomically related to members within the Family Dicistroviridae. The newly created family Dicistroviridae contains a single genus, Cripavirus. The type species of the genus Cripavirus is the cricket paralysis virus (CrPV) (Christian et al. 2000). Other species within this genus are: Drosophila C virus (DCV), Plautia stali intestine virus (PSIV), Himetobi P virus (HPV), Triatoma virus (TrV), black queen cell virus (BQCV), and Rhopalosiphum padi virus (RhPV). Tentative species within this genus include: acute bee paralysis virus (ABPV), and four other candidate species: aphid lethal paralysis virus (ALPV), Kashmir bee virus (KBV), cloudy wing virus (CWV) and Taura syndrome virus (TSV) (Mayo 2002). The use of viral capsid proteins have been shown to be a suitable target for phylogenetic studies in other insect viruses (Liljas et al. 2002, Tidona et al. 1998). As more insect-infecting viruses are sequenced and their genomic organization analyzed new virus relationships will be identified within the insect picorna-like viruses. Five clades were identified here: 1) DCV; 2) CrPV, 3) TrV, PSIV, HiPV, BQCV and HoCV-1; 4) ABPV, and KBV; and 5) RPV (Fig. 3). HoCV-1 had the highest matching BLASTP score (Table 1.) with Himetobi P virus, HiPV, which was discovered in planthoppers (Toriyama et al. 1992). Planthoppers are a taxonomically closely related insect group to leafhoppers within the Hemiptera. The virus was detected primarily in midgut tissues of H. coagulata, and this may be the primary site of infection and replication at least in leafhoppers.

Invading insects are constantly exposed to native pathogens as they move into new environmental niches. In this case, a leafhopper-infecting virus, HoCV-1, was identified that infects H. coagulata. Future research will need to focus on whether HoCV-1 can be used to reduce H. coagulata populations as a biological control agent, either by augmentation and release, or through other means such as bioengineering. Insect pathogens reduce populations naturally in the wild and if more individuals can be exposed to viral infections then the virus may ultimately be useful as a biological control measure (Hunter-Fujita et al. 1998). Many crops of economic importance such as grapes, almonds, pecans, and other woody tree crops, especially in California, and throughout the Southeastern part of the United States would benefit if leafhopper populations could be safely reduced. Further studies need to evaluate the mode(s) of virus transmission and stability in H. coagulata populations under field conditions. The largest obstacle with all viral pathogens is the development of a mass propagation system. Although cell cultures of H. coagulata have been established (Kamita et al. 2005) the ability for mass production of leafhopper viruses for commercial applications still remain unknown. The potential usefulness of HoCV-1 as a biological control agent against the H. coagulata and in reducing the spread of Pierce’s Disease, awaits the answers to these questions.
